# A feasibility study to develop and test a Spanish patient and provider intervention for managing osteoarthritis in Hispanic/Latino adults (PRIMO-Latino)

**DOI:** 10.1186/s40814-018-0280-x

**Published:** 2018-07-04

**Authors:** Leonor Corsino, Cynthia J. Coffman, Catherine Stanwyck, Eugene Z. Oddone, Hayden B. Bosworth, Ranee Chatterjee, Amy S. Jeffreys, Rowena J. Dolor, Kelli D. Allen

**Affiliations:** 1 Department of Medicine, Division of Endocrinology, Metabolism and Nutrition, Duke University Medical Center, Box 3451, Durham, NC 27710 USA; 2 Center for Health Services Research in Primary Care, Department of Biostatistics and Bioinformatics, Durham VA Medical Center, Duke University Medical Center, Duke Box 3827, Med Ctr, Durham, NC 27710 USA; 3 Durham VAMC, 508 Fulton St (152), Durham, NC 27705 USA; 4 Department of Medicine, Division of General Internal Medicine, Duke University Medical Center, Durham, NC USA; 5 Center for Health Services Research in Primary Care, Durham VA, Health Services Research, Durham VA Medical Center, Durham, NC 27705 USA; 6 Population Health Science Department, Professor in Psychiatry and Behavioral Science, Center for Health Services Research in Primary Care, Durham VA Medical Center, 411 West Chapel Hill Street, Suite 600, Durham, NC 27701 USA; 7 HSR&D (152) VA, 508 Fulton St, Durham, NC 27705 USA; 8 Department of Medicine, Division of General Internal Medicine, Duke University Medical Center, 411 West Chapel Hill Street, Suite 500, Durham, NC 27701 USA; 9 Center for Health Services Research in Primary Care, Durham VA Medical Center, 411 West Chapel Hill Street, Suite 600, Durham, NC 27701 USA; 10 Department of Medicine and Thurston Arthritis Research Center, University of North Carolina and Center for Health Services Research in Primary Care and Durham VA Medical Center, 3300 Thurston Building Campus Box 7280, Chapell Hill, NC 27599-7280 USA; 11 Center for Health Services Research in Primary Care, Durham VA Medical Center, 411 West Chapel Hill Street, Suite 600, Durham, NC 27701 USA

**Keywords:** Osteoarthritis, Arthritis, Hispanic, Behavior, Intervention, Spanish language, Primary care, Telephone intervention

## Abstract

**Background:**

Arthritis affects approximately 50 million adults in the USA. Hispanics/Latinos have a higher prevalence of arthritis-attributed activity limitations primarily related to osteoarthritis (OA). Hispanic/Latinos are less likely to receive hip replacement independent of health care access, and they are less likely to receive knee replacement. There have been few interventions to improve OA treatment among the Hispanic/Latino population in the USA. In our study, we aimed to develop and test a telephone delivered culturally appropriate Spanish behavioral intervention for the management of OA in Hispanic/Latino adults.

**Methods:**

We conducted a feasibility study in an academic health center and local community in Durham, North Carolina. We enrolled self-identified Spanish speaking overweight/obese adults (≥ 18) with OA of the knee and/or hip under the care of a primary health care provider. The 12-month patient intervention focused on physical activity, weight management, and cognitive behavioral pain management skills. The patient intervention was delivered via telephone with calls scheduled twice per month for the first 6 months, then monthly for the last 6 months (18 sessions). The one-time provider intervention included delivery of patient-specific OA treatment recommendations, based on patients’ baseline data and published guidelines. The primary measures were metrics of feasibility, including recruitment and intervention delivery. We also assessed pain, stiffness, and function using the Spanish-Western Ontario and McMaster Universities Osteoarthritis Index (WOMAC).

**Results:**

A total of 1879 participants were identified for potential enrollment. Of those, 1864 did not meet inclusion criteria, were not able to be reached or refused. Fifteen participants enrolled in the intervention. The mean number of phone calls completed was 14.7. Eighty percent completed more than 16 calls. The mean WOMAC baseline score (SD) was 39 (20); mean improvement in WOMAC scores between baseline and 12 months, among 11 participants who completed the study, was − 13.27 [95% CI, − 25.09 to − 1.46] points.

**Conclusion:**

Recruitment of Hispanics/Latinos, continues to be a major challenge. A Spanish-based telephone delivering lifestyle intervention for OA management in Hispanic/Latino adults is feasible to deliver and may lead to improved OA symptoms. Future research is needed to further test the feasibility and effectiveness of this type of intervention in this segment of the population.

**Trial registration:**

NCT01782417

## Background

Arthritis affects approximately 50 million adults in the USA, making it one of the most common causes of disability in this county [1]. Arthritis is associated with significant physical activity limitations, increased prevalence of obesity, decreased health related quality of life, and increased health care costs [1, 2]. Hispanics/Latinos, the largest minority population in the USA, are affected by arthritis at a slightly lower age-adjusted rate compared to non-Hispanic whites and African Americans. However, Hispanics/Latinos have a higher prevalence of arthritis-attributed activity limitations (primarily related to osteoarthritis (OA)) [1]. Hispanic/Latinos are less likely to receive hip replacement independent of health care access, and they are less likely to receive knee replacement [3–5]. This highlights the importance of reaching Hispanics/Latinos with interventions that improve arthritis-related care and outcomes. Evidence-based guidelines emphasize the importance of the combination of medical and behavioral modalities for treating OA, especially in the earlier stages [6, 7]. However, there have been few interventions developed or studied to improve OA treatment particularly for the Hispanic/Latino population in the USA.

In addition, methods for improving access to OA interventions are lacking. To our knowledge, a telephone-based delivered patient intervention for managing OA has not been tested in Hispanics/Latinos. Telephone-based interventions have the capability of reaching large numbers of patients at relatively low cost, without the time and transportation barriers usually faced by Hispanics/Latinos when engaging in in-person programs [8]. However, we need to understand whether a telephone-based format of this type of program is feasible and potentially effective for Hispanics/Latinos with OA. In this manuscript, we report the results of a feasibility study testing a telephone-delivered culturally appropriate Spanish behavioral intervention for the management of OA in Hispanic/Latino adults.

## Methods

### Data source and study design

PRIMO-Latino was designed with two main objectives:(1) to develop culturally appropriate Spanish materials for a patient OA intervention (involving exercise, weight management, and cognitive behavioral pain management) and a provider-based intervention (involving provision of patient-specific recommendations for care) and (2) to test the Spanish patient-based intervention among Hispanic/Latino adults with OA. This study was conducted in parallel with the Patient and Provider Interventions for Managing Osteoarthritis in Primary Care (PRIMO) [9, 10]. PRIMO-Latino was a single group feasibility study conducted in the Duke Health System primary care clinics, the Duke Center for Living -*Sarah* W. *Stedman* Nutrition and Metabolism Center in Durham North Carolina, and a local church. All participants received the intervention. The study was approved by Duke University Medical Center Institutional Review Board. All participants provided written informed consent in Spanish.

### Participants

We aimed to enroll 25 participants following the same recruitment approach utilized for enrollment in the parent study [9, 10] However, due to a large number of participants not fulfilling inclusion criteria, specifically no diagnosis of OA and without primary care follow up at Duke and/or lack of a primary care provider, we enrolled a total of 15. Inclusion criteria were as follows: diagnosis of hip OA (based on radiographic evidence in the electronic medical record) and/or knee OA (based on radiographic evidence in the electronic medical record or meeting American College of Rheumatology clinical criteria) [11], current symptoms in the joint(s) with OA, body mass index (BMI) ≥ 25, older than 18 years, self-identification as Hispanic/Latino, not currently meeting Departments of Health and Human Services physical activity recommendations [12], Spanish speaker, and under the care of a health care provider per participant report (one visit within the last 12–18 months). Key exclusion criteria were other rheumatologic conditions, hip or knee surgery or acute meniscus or anterior cruciate ligament tear in the past 6 months, recent hospitalization for cardiovascular/cerebrovascular event, serious mental health conditions, on waiting list for hip or knee arthroplasty, motor neuron diseases, terminal illness, and current participation in another OA intervention or lifestyle change study.

### Recruitment procedures

Similar to the PRIMO study, we identified potential participants using the Duke electronic medical records. We identified patients who had ICD-9 codes for knee/hip OA (715.xx) and knee/hip pain (719.xx). We expanded the codes to include pain in a limb (729.5) since relatively few Hispanic/Latino patients were identified who had OA-specific codes. Following these data pulls (*n* = 3), the team reviewed patients’ records to confirm eligibility. Also, we presented our study to primary care clinics in the Duke Health System and at local free clinics. A total of 1840 charts were reviewed. Participants meeting inclusion criteria received an introductory letter in Spanish by mail, signed by the patient’s primary care provider. Those who met inclusion criteria received a screening telephone call to further assess and confirm eligibility (including being a Spanish speaker, since not all indviduals with Hispanic/Latino ethnicity noted in the electronic medical record are Spanish speakers).

Due to a low rate of patients meeting eligibility criteria, we expanded our recruitment efforts by presenting our study at local Hispanic/Latino churches and events and distributed flyers in the local free clinics and Hispanic/Latino-serving clinics in the area. Those expressing interest at local events and those referred directly by primary care providers received a follow-up call to assess eligibility. Patients meeting criteria met a study member at their clinic site, church or at the Duke *Sarah* W. *Stedman* Nutrition and Metabolism Center. A Hispanic/Latino native Spanish speaker reassessed clinical criteria, as well as height and weight to determine body mass index (BMI). After confirming eligibility criteria, participants signed an informed consent.

#### Interventions

The interventions mirrored the PRIMO study interventions [9]. All materials were adapted to reflect the cultural/language diversity within the Hispanic/Latino population. The patient education book was developed with a local organization with expertise in developing research materials for diverse populations. The cultural adaptation of the intervention focused on language utilized by different countries in Latin America and included photos, in the patient education material of Hispanics/Latinos that represented the racial diversity of this population. All materials were reviewed for language, content, and how the materials reflected their culture prior to the conduct of the study, by a total of five Hispanics/Latinos: one each from Dominican Republic, Mexico, Colombia, Puerto Rico, and mixed Dominican and Honduras descent. All reviewers agreed that the material was culturally appropriate, and suggestions predominantly focused on improving the Spanish language of the material.

The 12-month patient intervention focused on physical activity for patients with OA, weight management, and cognitive behavioral pain management. The intervention was delivered via telephone, in Spanish, by a native Spanish speaker physician. Calls were scheduled twice per month for the first 6 months and then monthly for the last 6 months. The interventionist was flexible with nights and occasional weekends to facilitate participation. The interventionist focused on delivering targeted educational content, as well as goal setting and action planning. The first 3 months, participants choose to focus on either weight management or physical activity; the other topic was covered for the second 3 months. This allowed consistency in delivery of all educational content surrounding weight management and physical activity while providing participants a choice regarding the order in which the content was presented. The final 6 months focused on participants’ goals related to physical activity and weight management. Cognitive behavioral pain management skills were discussed throughout the intervention. Participants received patient education book, CDs developed for physical activity and relaxation techniques, and a therapy/exercise band.

The provider intervention was limited to delivering patient-specific OA treatment recommendations, based on published guidelines [7] and tailored based on participants’ baseline information. The recommendations included non-pharmacological and pharmacological therapies based on algorithms developed for the PRIMO study [9, 10]. We intended to use electronic medical records to deliver the provider recommendations. However, because only six participants had a Duke Health Care Provider, we instead provided participants a letter, directed to their self-reported primary care provider that included the patients’ specific recommendations. Patients were encouraged to give the letter to their provider during their next visit.

### Measures

#### Feasibility outcomes

We collected data on numbers of (a) potentially eligible participants obtained from electronic medical record data pull, (b) self-referred potentially eligible participants, (c) enrolled participants, (d) participants completing the phone calls and the intervention.

#### Outcome measures

The primary outcome was the Spanish-Western Ontario and McMaster Universities Osteoarthritis Index (WOMAC), a self-reported measure of lower-extremity pain (5 items), stiffness (2 items), and function (17 items) in the past 2 weeks [13] administered at baseline, 6 and 12 months. Items are rated on a 5-point Likert scale ranging from “none” to “extreme” (total range, 0 to 96; higher scores indicate worse symptoms and function). WOMAC and BMI were collected at baseline and 12 months in person and at 6 months via telephone. We administered the Short Physical Performance Battery (SPPB) [14], which includes 3 tests of balance, a timed 8-ft walk, and 5 chair stands. The total depressive symptoms were assessed with the Spanish version of the Patient Health Questionnaire (PHQ-8); [15] an 8-item questionnaire with scores ranging from 0 to 24 at baseline and 12-month follow-up.

#### Demographic and clinical characteristics collected at baseline

Age (calculated from the date of birth), gender, educational level, marital status, country of origin, years living in the USA, self-reported income, work status, and health insurance. Self-reported general health (excellent, very good, good or fair, poor), BMI, the presence of knee OA, hip OA or both (determined as described above for inclusion criteria), and self-reported duration of OA.

#### Analysis

Only descriptive statistics are presented due to small sample size and a lack of statistical power. Baseline to 6 and 12-month change scores were calculated for WOMAC total score, WOMAC pain and function subscales and BMI. We calculated, change in the SPPB and the PHQ-8 from baseline to 12 months. Means, standard deviations (SD), and 95% confidence intervals are presented.

## Results

### Participants

**Fig. 1:**
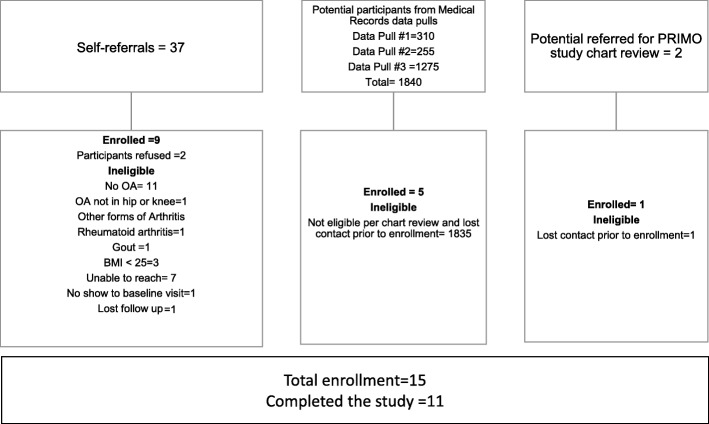
Source of participants enrollment

**Fig. 2:**
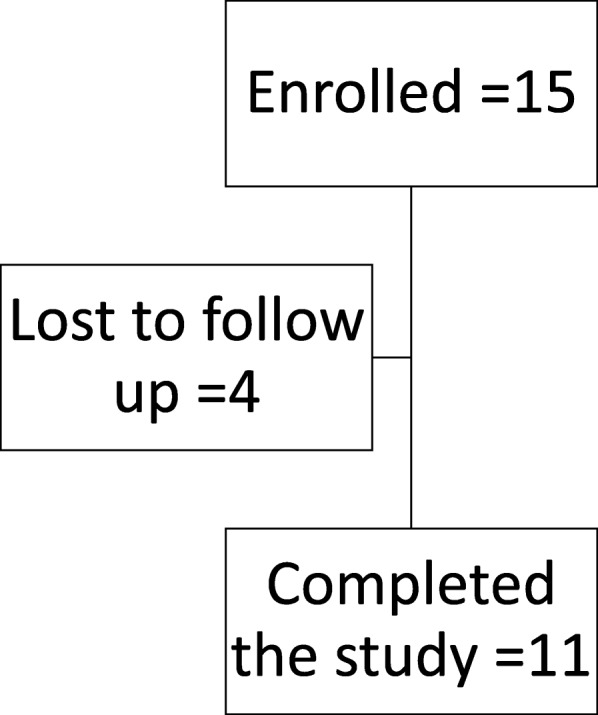
Participants flow

We identified 1840 potentially eligible participants using the Duke Electronic Medical Record, 37 from self-referral and 2 from PRIMO study referral (Fig. 1). Of those, 15 met inclusion criteria and enrolled in the study. Twelve participants had follow-up at 6 months, and 11 had follow-up at 12 months. Four participants were lost during follow-up at 6 and/or 12 months (Fig. 2).

### Intervention delivery

Mean number of completed phone calls was 14.7 (SD = 0.7) out of 18 (Range 5–19). One participant requested an additional phone call. Twelve (80%) completed more than 16 calls. Baseline characteristics in Table 1.

**Table 1:** Participants baseline characteristics

Characteristic	Total sample (*n* = 15)^a^
Age in years, mean (SD)	46 (11)
Women, *n* (%)	13 (87)
High school education or less, *n* (%)	11 (74)
Married, *n* (%)	8 (53)
Country of origin, *n* (%)	
Mexico	7 (47)
Ecuador	1 (6.7)
Honduras	5 (33.3)
Panama	1 (6.7)
Puerto Rico	1 (6.7)
Years in the USA, mean (SD)	15.4 (6.5)
Income < $30,000 per year per family, *n* (%)	9 (60)
Work status, *n* (%)^a^	
Full time	4 (27)
Part time	6 (40)
Unemployed	3 (20)
No health insurance, *n* (%)	11 (74)
BMI, mean (SD), kg/m^2^	32 (7)
Joints with osteoarthritis, *n* (%)	
Knee only	11 (73.3)
Hip only	1 (6.7)
Knee and hip	3 (20)
Duration of arthritis symptoms, mean (SD), years	6.3 (7)

^a^Some measurements had missing values

*BMI* body mass indexm, *WOMAC* Spanish-Western Ontario and McMaster Universities Osteoarthritis Index, *SD* standard deviation, *SPPB* short physical performance battery, *PHQ-8* 8-item Patient Health Questionnaire

### Changes in outcomes

Baseline mean WOMAC score (SD) for the 15 enrolled participants was 38.6 (19.1). Mean WOMAC score (SD) for the 11 participants with baseline and 12 months follow-up was 41.5 (20.1) Mean change in WOMAC scores between baseline and 6 months for the 12 participants with 6-month follow-up was − 18.4 points [95% CI − 27.8 to − 9.0]. Mean change in WOMAC scores between baseline and 12 months for the 11 participants with 12 months follow-up was − 13.3 points [95% CI, − 25.1 to − 1.5]. Mean changes for WOMAC pain and physical function between baseline and 6 months were − 4.3 [95% CI, − 7.4 to − 1.3] and − 12.1 [95% CI, − 18.2 to − 6.0], respectively. Mean change in WOMAC pain and physical function between baseline and 12 months were − 3.6 [95% CI, − 7.1 to − 0.0] and − 8.1 [95% CI, − 16.5 to 0.3]. A negative change in the WOMAC score indicates improvement. Mean change in BMI at 6 and 12 months were − 1.1 [95%CI, − 1.9 to − 0.2] and − 0.5 [95% CI, − 1.6 to 0.6], respectively. Mean change in the SPPB at 12 months was 2.9 [95% CI, 0.7 to 5.0] and the PHQ-8 was − 2.0 [95% CI, − 6.2 to 2.2] Table 2.

**Table 2:** Observed mean, mean change and 95% CI for WOMAC, WOMAC pain and physical function, BMI, SPPB, and PHQ-8 by time point

Outcomes	*N*	Mean baseline	*N*	Missing	Baseline to 6-month difference (95% CI)	*N*	Missing	Baseline to 12-month difference (95% CI)
WOMAC	15	41.5	11	4	− 18.4 (− 27.8 to − 9.0)	11	4	− 13.3 (− 25.1 to − 1.5)
WOMAC pain	15	9.5	11	4	− 4.3 (− 7.4 to − 1.3)	11	4	− 3.6 (− 7.1 to − 0.0)
WOMAC physical function	15	27.7	11	4	− 12.1 (− 18.2 to − 6.0)	11	4	− 8.1 (− 16.5 to 0.3)
BMI^a^	15	32.1	11	4	− 1.1 (− 1.9 to − 0.2)	11	4	−0.5 (− 1.6 to 0.6)
SPPB^b^	13	6.8	NA	NA	NA	7	8	2.9 (0.7 to 5.0)
PHQ-8^b^	15	7.7	NA	NA	NA	11	4	−2.0 (−6.2 to 2.2)

^a^Calculated with self- reported weight at 6 months

^b^Not measured at 6 months

*WOMAC* Spanish-Western Ontario and McMaster Universities Osteoarthritis Index, BMI, body mass index, *SPPB* short physical performance battery, *PHQ-8* 8-item Patient Health Questionnaire, *NA* not available

## Discussion

### Summary of main findings and comparison with previous studies

We successfully developed a culturally appropriate Spanish patient and provider osteoarthritis (OA) interventions and delivered the intervention by phone among Hispanic/Latino adults with OA. We successfully developed the intervention materials so they were appropriate for a diverse Hispanic/Latino population and demonstrated that a phone delivered intervention can be potentially effective. The engagement was high among those enrolled in the study; 12 (80%) completed more than 16 out of 18 intervention calls. This provides evidence of the acceptability of this type of intervention among Hispanic/Latino patients with OA. Further, participants who completed the study showed a change in the WOMAC score of − 13.3, which is a clinically meaningful 32% improvement in patients’ OA symptoms. This difference was larger than what we saw in the parent study [9]. The changes in WOMAC total and subscale scores were somewhat larger at 6 months than at 12 months. One possible reason is that intervention call frequency was reduced after 6 months. It is possible that continued more frequent calls would help participants to sustain greater benefits. Further, in our study, similar to other lifestyle intervention programs, our participants showed a relatively small improvement in their weight and BMI at 6 months, with some weight regain by 12 months [16, 17]. We can speculate that the reason for this small decrease in weight and BMI is likely due to the fact that the internsivity of the weight loss component of the intervention was not enough to yield clinically meaningful weight reduction. Also, we achieved improvement in the SPPB and PHQ8.

### Strengths, challenges, and weaknesses

We wanted to determine the feasibility of delivering the intervention. We enrolled a diverse group of Hispanic/Latino with different cultural backgrounds. However, we faced several challenges recruiting participants and were not able to meet our enrollment goal. A major challenge was our inability to enroll participants with an established primary care provider. Initially, we aimed to enroll participants from our health system so that we could deliver the provider intervention via the electronic medical record as in PRIMO study. However, despite identifying a large number of potential participants and after reviewing a large number of patients’ records, the vast majority did not meet our eligibility criteria including OA diagnosis, no primary care provider, no visit within the specified time frame, etc. A potential explanation for a lack of not meeting our inclusion criteria included the fact that the local Hispanic/Latino population is relatively young, recent immigrants and a large proportion are without health insurance. In addition, a significant portion of our local Hispanic/Latino population does not have an established primary care and received care from the local Federally-Qualified Health Center and the area free clinics. We did not collect rigorous qualitative data from participants regarding recruitment and retention challenges; this is a limitation and would be beneficial for future studies of this type.

Additional limitations included, first, we were not able to meet our recruitment goals. Second, our lack of comparison group prevents strong conclusions on the impact of the intervention on outcomes. Third, we were not able to follow up to assess the impact of the intervention on the provider side of the intervention because a total of nine participants had providers outside our health system.

Our study also has significant strengths. First, we were able to develop a culturally sensitive intervention for the management of OA in Hispanics/Latinos something that, to our knowledge, has not been done. This is important because this is a population that faces a significant amount of health disparities including OA management. Second, we enrolled a diverse cohort of Hispanics/Latinos representing different cultures and countries within the Hispanic/Latino population. Third, we were able to show improvement in multiple outcomes by the end of the intervention. Lastly, 80% of the participants completed more than 16 calls of the intervention indicating that a telephone approach is potentially better for reaching this population than face-to-face interventions.

## Conclusion

Lack of continuity of care and access to health care remains a major limitation in the adequate treatment of osteoarthritis for the Hispanic/Latino population. In this pilot study, we tested the feasibility of recruitment and delivery of a telephone-based intervention for the treatment of OA in Hispanic/Latino adults. Despite our recruitment challenges and small sample size, our pilot study shows that an intervention delivered by telephone for the treatment of OA in Hispanic/Latino adults had benefit among our participants, with high engagement with the intervention and a clinically relevant improvement in osteoarthritis symptoms. Future research is needed to improve recruitment into clinical trials of Hispanics/Latinos and to further test the effectiveness of this type of intervention in this population.
